# Rapidly Enlarging Intra-abdominal Ileal Duplication Cyst in a Newborn

**Published:** 2016-04-24

**Authors:** Emrah Aydın

**Affiliations:** Department of Pediatric Surgery, Bahcelievler State Hospital, Turkey

**Dear Sir,**

An 8-day old girl, born via vaginal delivery and healthy otherwise, was admitted with the antenatal diagnosis of an intra-abdominal cyst, 5mm in diameter at 30th gestational week. Abdominal ultrasonography (USG) was performed on first postnatal day showed a 10mm cyst originating from right ovary. She remained asymptomatic for a week when she developed bilious vomiting. Her physical examination was normal except for a palpable mass on the right side of the abdomen. X-ray abdomen showed bowel loops displaced to left half of the abdomen. Repeat USG showed an increase in size of cyst to 49mm. Six hours later another USG was performed owing to further increase in abdominal distension. The size of cyst increased to 80x70x40mm moreover debris was also noted in it. The baby however remained vitally stable. Next day another USG showed cyst size of 100x80x45mm. Because of increase in the diameter of cyst and bilious drainage from nasogastric tube, laparoscopy was performed. This showed normal looking ovaries. The procedure was then converted to laparotomy. At exploration a communicating ileal duplication cyst was found (Fig. 1). Meckel’s diverticulum was also present 50cm away from the cecum. Resection of the cyst and anastomosis was performed. Appendectomy was also done but Meckel’s diverticulum was left as such. Postoperative course was uneventful. Histopathology was reported as ileal duplication cyst. She is asymptomatic for the last one year.

**Figure:**
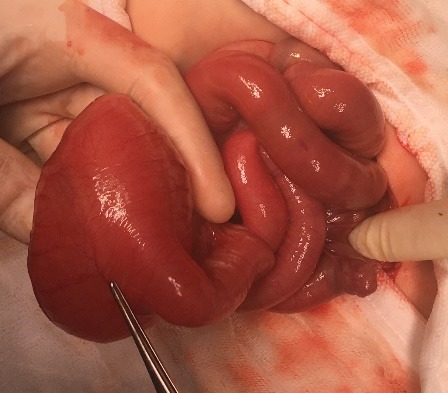
Figure 1: Peroperative view of cyst.

Alimentary tract duplications are very rare congenital malformations with reported incidence of 1 in 18000 live births.[1, 2] Ileum is most common location of these cysts. In our case patient had prenatal diagnosis of intra-abdominal cyst suspected of ovarian origin. The unusual postnatal feature was continuous and rapid increase in the size of the cyst with bilious vomiting. This was due to the communication of cyst with the adjacent ileum the contents of which pooled in the cyst. This coupled with mucoid secretions of the cyst resulted in rapid increase in the size.

## Footnotes

**Source of Support:** Nil

**Conflict of Interest:** None declared

